# Pivotal Importance of STAT3 in Protecting the Heart from Acute and Chronic Stress: New Advancement and Unresolved Issues

**DOI:** 10.3389/fcvm.2015.00036

**Published:** 2015-11-30

**Authors:** Fouad A. Zouein, Raffaele Altara, Qun Chen, Edward J. Lesnefsky, Mazen Kurdi, George W. Booz

**Affiliations:** ^1^American University of Beirut Faculty of Medicine, Beirut, Lebanon; ^2^Department of Pharmacology and Toxicology, School of Medicine, The University of Mississippi Medical Center, Jackson, MS, USA; ^3^Division of Cardiology, Department of Internal Medicine, Pauley Heart Center, Virginia Commonwealth University, Richmond, VA, USA; ^4^Department of Biochemistry and Molecular Biology, Virginia Commonwealth University, Richmond, VA, USA; ^5^McGuire Department of Veterans Affairs Medical Center, Richmond, VA, USA; ^6^Department of Chemistry and Biochemistry, Faculty of Sciences, Lebanese University, Hadath, Lebanon

**Keywords:** mitochondria, U-STAT3, complex I, myocardial infarction, cardiac hypertrophy, heart failure, peripartum cardiomyopathy, ischemic pre- and post-conditioning

## Abstract

The transcription factor, signal transducer and activator of transcription 3 (STAT3), has been implicated in protecting the heart from acute ischemic injury under both basal conditions and as a crucial component of pre- and post-conditioning protocols. A number of anti-oxidant and antiapoptotic genes are upregulated by STAT3 *via* canonical means involving phosphorylation on Y705 and S727, although other incompletely defined posttranslational modifications are involved. In addition, STAT3 is now known to be present in cardiac mitochondria and to exert actions that regulate the electron transport chain, reactive oxygen species production, and mitochondrial permeability transition pore opening. These non-canonical actions of STAT3 are enhanced by S727 phosphorylation. The molecular basis for the mitochondrial actions of STAT3 is poorly understood, but STAT3 is known to interact with a critical subunit of complex I and to regulate complex I function. Dysfunctional complex I has been implicated in ischemic injury, heart failure, and the aging process. Evidence also indicates that STAT3 is protective to the heart under chronic stress conditions, including hypertension, pregnancy, and advanced age. Paradoxically, the accumulation of unphosphorylated STAT3 (U-STAT3) in the nucleus has been suggested to drive pathological cardiac hypertrophy and inflammation *via* non-canonical gene expression, perhaps involving a distinct acetylation profile. U-STAT3 may also regulate chromatin stability. Our understanding of how the non-canonical genomic and mitochondrial actions of STAT3 in the heart are regulated and coordinated with the canonical actions of STAT3 is rudimentary. Here, we present an overview of what is currently known about the pleotropic actions of STAT3 in the heart in order to highlight controversies and unresolved issues.

## Introduction

**Figure 1:**
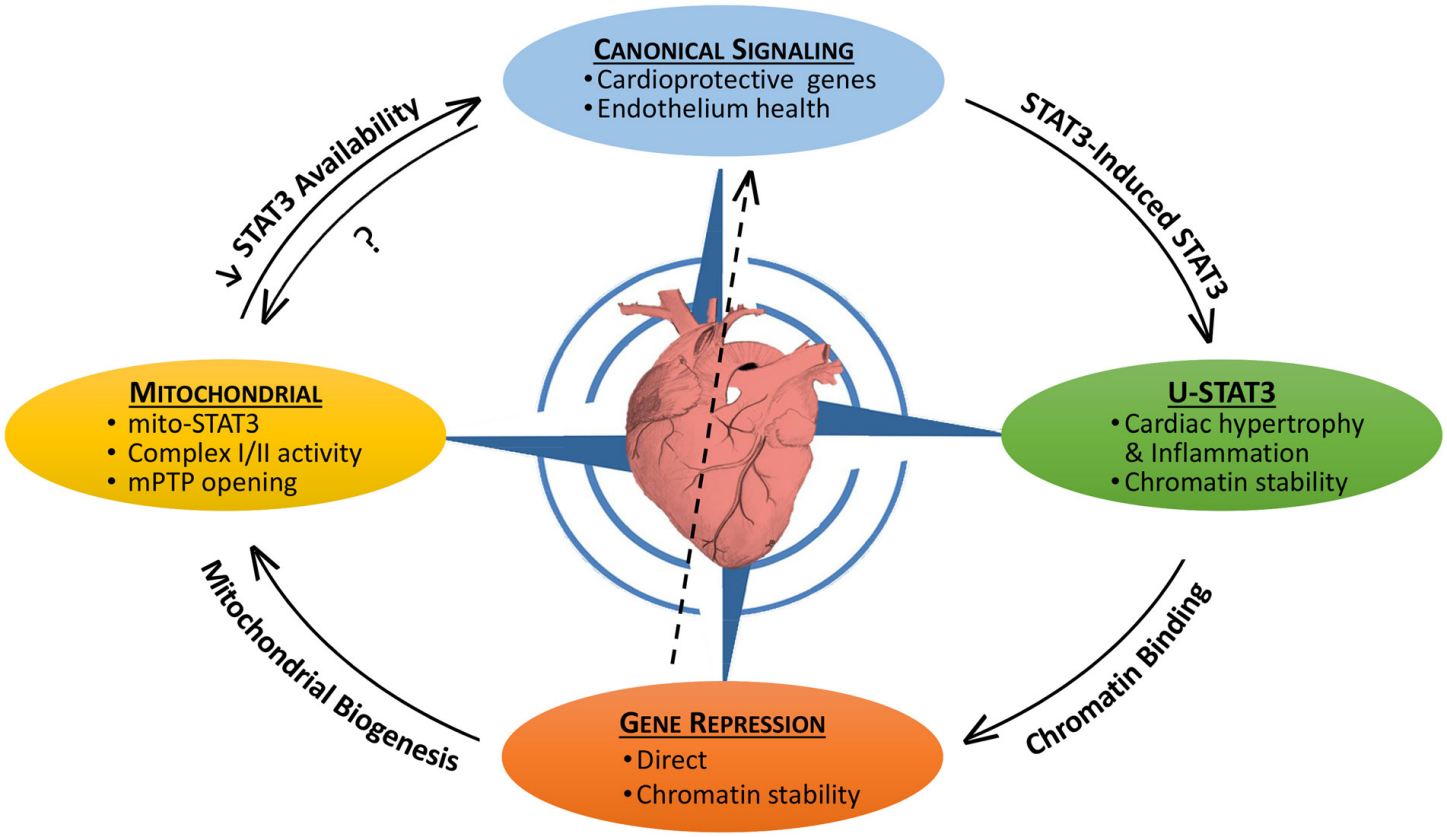
**The pleotropic actions of STAT3 in the heart and their interplay**. STAT3 has both canonical and non-canonical actions that impact on the viability of the myocardium. Canonical STAT3-induced gene expression is associated with the upregulation of protective anti-oxidant and anti-apoptotic proteins, as well as maintaining the heath of the endothelium (e.g., by upregulating VEGF and SOD2). STAT3 induces its own expression leading potentially to the accumulation of unphosphorylated STAT3 (U-STAT3) in the nucleus, which has been associated with hypertrophy and inflammation. U-STAT3 may contribute to chromatin stability and gene suppression. STAT3 also directly suppresses/represses genes possibly by association with DNMT1 or HDACs. Loss of STAT3-mediated suppression of miR-199a-5p has been linked to endothelial dysfunction (center arrow). Gene repression may be important for mitochondrial biogenesis. STAT3 has direct mitochondrial actions that are poorly understood, but involve maintaining complex I/II activity and preventing mPTP opening. By sequestering STAT3 (perhaps involving redox modifications), mitochondria may reduce the nuclear transcriptional actions of STAT3. Canonical STAT3 signaling is likely important for mitochondrial health as well.

Signal transducer and activator of transcription 3 (STAT3) is both a transcription factor and signaling molecule that modulates mitochondrial function (1–5). In the heart, both the protein levels and activation status of STAT3 are dynamic, as is its subcellular distribution. In this context, STAT3 acts as a sensor of both acute and chronic stress so as to implement protective measures against oxidative damage or pro-death signaling, or perhaps ensure structural stability of microtubules or chromatin (1). Notably, STAT3 levels and activity in the heart are reported to be reduced with heart failure, aging, and diabetes (1, 6–11). In the nucleus, STAT3 as a transcription factor has both canonical actions, very often in association with NF-κB, which are initiated by external stimuli and non-canonical actions that result from its accumulation due to a STAT3-induced STAT3 circuit (1). STAT3’s non-canonical genomic actions may include as well modulation of chromatin structure (1). In addition, STAT3 has non-genomic (non-canonical) actions involving regulation of mitochondrial function and integrity. Our understanding of the interplay between the canonical and non-canonical genomic and non-genomic, actions of STAT3 within the stressed or diseased heart is still fairly basic (Figure 1).

Signal transducer and activator of transcription 3 is a transcription factor that is activated by phosphorylation on a specific tyrosine residue (Y705) in cardiac myocytes by agonists that engage membrane receptors directly coupled to activation of Just Another Kinase (JAK) 1 or JAK2 tyrosine kinases (1–5). These agonists have pathophysiological importance and include the interleukin 6 (IL-6) family cytokines, such as IL-6 and leukemia inhibitory factor (LIF), tumor necrosis factor (TNF), interferon gamma (IFNγ), erythropoietin, and granulocyte-colony stimulating factor (G-CSF). Y705 can also be phosphorylated by Src family kinases and tyrosine kinase bone marrow kinase on chromosome X (BMX) in cardiac myocytes (12–14), although the physiological significance of this is unclear. Mechanical stretch and pressure overload, but not volume overload, are reported to activate STAT3 in cardiac myocytes or the myocardium (13, 15–22). Pressure overload-induced activation of STAT3 in the heart is mainly dependent on the autocrine/paracrine-release of IL-6 family cytokines, with a lesser contribution from local angiotensin II production (19–22). Angiotensin II activates STAT3 in cardiac myocytes and fibroblasts directly *via* the angiotensin II type 1 (AT1) receptor, which contains a JAK2 binding site in the C-terminus, as well as by upregulating expression of IL-6 family cytokines (23–25).

Besides functioning as a transcription factor STAT3 is now known to have poorly understood non-genomic actions in mitochondria that modulate respiration, reactive oxygen species (ROS) formation, and opening of the mitochondrial permeability transition pore (mPTP) (1, 4, 5). Overwhelming evidence supports the conclusion that STAT3 is important for the protection of the heart from acute ischemic stress by both genomic and non-genomic means (1). Although less well studied, STAT3 appears to be important for protection of the heart from chronic stress, such as pressure overload (17). We also observed that mice homozygous for a STAT3 S727A mutation that impairs both genomic and non-genomic actions exhibited cardiac dysfunction and evidence of cardiac myocyte necrosis at an early stage of angiotensin II-induced hypertension (26). In this review, we present an overview of the role of STAT3 in the heart in acute and chronic stress with a focus on unresolved issues and controversies.

### Posttranslational Modifications of STAT3

**Figure 2:**
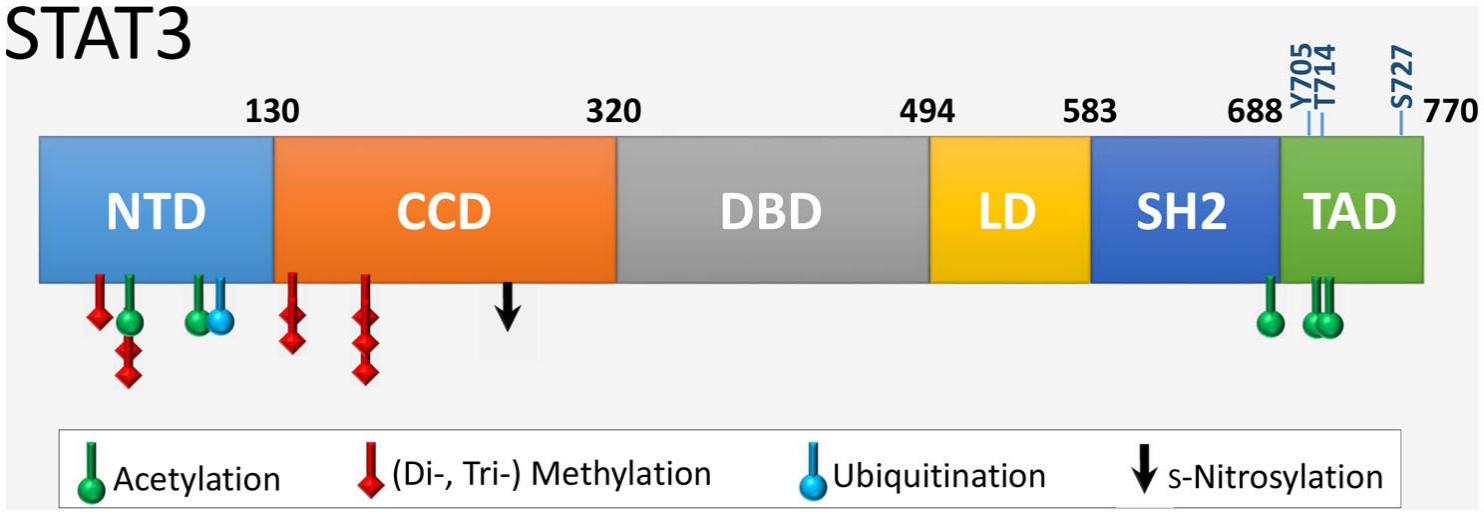
**The six functional domains of STAT3**. NTD, NH_2_-terminal domain; CCD, coiled coil domain; DBD, DNA-binding domain; LD, linker domain; SH2 domain; TAD, transcription activation domain. The location of residues that are targets of various posttranslational modifications are indicated. Shown also are the two key regulatory residues by phosphorylation (Y705 and S727) within the TAD, as well as the novel site of phosphorylation T714 linked to transcriptional activity.

Signal transducer and activator of transcription 3 is 770 amino acids in length with six distinct domains (Figure 2). The coiled coil domain is involved in protein–protein interactions, and the SH2 domain mediates STAT3 dimerization *via* intermolecular phosphorylated tyrosine–SH2 interactions. The amino acid sequence of STAT3 is highly conserved across species. STAT3 is modified at specific residues by a number of posttranslational modifications with functional consequences, most notably by phosphorylation and acetylation (Table 1). In addition, STAT3 can undergo s-nitrosylation, s-glutathionylation, di- or trimethylation, and mono-ubiquitination, although these modifications have not been specifically demonstrated in cardiac cells.

**Table 1:** **Posttranslational modifications of STAT3**.

Residue	Domain	Modification	Function
R31	NTD	Methylation	• Attenuation of activation (27)
K49	NTD	Dimethylation	• Increased gene expression (28)
K49, K87	NTD	Acetylation	• Stable p300 association and gene expression (29, 30)
			• HDAC1 association and nuclear export (termination of transcription) (31)
K87	NTD	Acetylation	• Sin3a-mediated transcription repression (32)
K97	NTD	Mono-ubiquitination	• Anti-apoptotic (BRD4-dependent) gene expression (33)
K140	CCD	Dimethylation	• Decreased gene expression (34)
K180	CCD	Trimethylation	• Increased Y505 phosphorylation/gene expression (35)
C259	CCD	S-nitrosylation	• Inhibits Y705 activation (phosphorylation) (36)
K685, K707, K709	SH2, TAD, TAD	Acetylation	• Regulation of Y705 phosphorylation (37)
			• Inhibition of gluconeogenesis genes in liver (37)
K685	SH2	Acetylation	• Stable dimer formation, DNA binding, transcription (38, 39)
			• Interaction with DNMT1 and gene silencing (40, 41)
			• U-STAT3-mediated gene expression (42)
Y705	TAD	Phosphorylation^a^	• Enhances dimerization and transcription/gene expression (1)
T714, S727	TAD	Phosphorylation	• Gene expression (43)
S727	TAD	Phosphorylation^a^	• Enhances gene expression (44)
			• Mitochondrial actions (1, 4, 5, 45–48)
			• Recruitment of tyrosine phosphatases/transcription termination (1, 49, 50)

*CCD, coiled coil domain; NTD, N terminus domain; SH2, Src homology 2 domain; TAD, transcription activation domain. ^a^Demonstrated in cardiac myocytes*.

#### Phosphorylation

Two sites of phosphorylation are important in canonical STAT3 activation and gene expression and are located in the C-terminal regulatory transcription activation domain or TAD (1). Phosphorylation of Y705 favors formation of parallel STAT3 dimers that translocate to the nucleus and induce expression of certain genes containing an interferon γ (gamma)-activated sequence (GAS) element (TTCNNNGAA or variation thereof, such as a *sis*-inducible element, SIE) in their promoter (4, 23). The other site is S727, which is phosphorylated in the cytoplasm or nucleus by several serine/threonine kinases, including ERK1/2, protein kinase Cϵ (PKCϵ), PKCδ, ZIP kinase, mTOR, and CDK5 (1). In canonical STAT3 signaling S727 phosphorylation boosts the transcriptional activity of STAT3 by recruiting histone acetyltransferase p300/CBP (44). Phosphorylation of S727 is also important for interaction of STAT3 with GRIM19 (complex I subunit B16.6 or NDUFA13) and uptake of STAT3 by mitochondria (45, 51). The TAD of STAT3 has other functions as well (1). Of note, recent evidence suggests that Y705 phosphorylation may not be obligatory for canonical STAT3 signaling and gene expression. In endothelial cells in response to simultaneous activation of the epidermal growth factor receptor (EGFR) and protease-activated receptor 1 (PAR-1), phosphorylation of STAT3 at T714 and S727 by glycogen synthase kinase (GSK) 3α and β was linked to gene expression in the absence of Y705 phosphorylation (43).

#### Acetylation

Acetylation of STAT3 on several lysine residues by p300 is an essential determinant of its transcriptional activity. Acetylation of K49 and K87 in the NH_2_ terminus is required for p300 binding and gene expression, although inducible DNA binding is not affected (29, 30). In addition, the NH_2_-terminal acetylation domain is required for HDAC1 binding and termination of transcription by STAT3 (31). Thus, mutation of these lysine residues delays subsequent redistribution of STAT3 from the nucleus. Acetylation of K685 in the SH2 domain may represent the initial contact of p300 with STAT3 (29–31, 38). K685 acetylation is reported to enhance DNA binding, transactivation activity, and nuclear localization of STAT3 (38, 39), although the conclusion that K685 is important for stable STAT3 dimer formation has been questioned based on structural considerations (52). Recently, evidence was provided that K685 acetylation is more important for gene expression by unphosphorylated STAT3 (U-STAT3), rather than in response to tyrosine-phosphorylated STAT3 (42). K685 acetylation has also been implicated in gene silencing by STAT3 *via* the targeting of the DNA methyl transferase, DNMT1 to certain promoters (40, 41). Binding of STAT3 to DNMT1 is regulated by K685 acetylation of STAT3 by p300 (40).

Other lysine residues of STAT3 are likely targets of acetylation with functional consequences. For instance, repression of STAT3 transcriptional activity by the histone deacetylase Sin3a is reported to be dependent on K87 acetylation as the main regulator of STAT3–Sin3a interaction (32). In the liver, STAT3-mediated inhibition of gluconeogenesis by gene suppression during the fed state was found to be regulated by a cluster of lysine residues (K679, K685, K707, and K709) in the C-terminus (37). Of particular note, Y705 phosphorylation and activation of STAT3 was found to be dependent upon acetylation of these residues and opposed during the fasting state by SIRT1. The basis for the positive role of multiple C-terminal acetylation sites on STAT3 activation and whether a similar scenario occurs in the heart awaits investigation.

#### Methylation and Mono-Ubiquitination

Posttranslational modification of various NH_2_-terminal lysine residues by methylation is a recently described mechanism that regulates STAT3’s transcriptional activity (28, 34, 35). Although reported thus far for cancer cells, lysine methylation is likely a basic transcriptional event important in cardiac cells as well. At least for K49 and K140, methylation occurs after STAT3 Y705 phosphorylation and binding to certain promoters (28, 34, 53). Dimethylation of K49 and K140 by the histone modifying enzymes EZH2 and SET9, respectively, was shown to be important in regulating expression of many IL-6-dependent genes in colon A4 cancer cells (28, 34). Dimethylation of K49 and K140 was associated with increased and decreased gene expression, respectively. Trimethylation of K180 by EZH2 was reported to enhance STAT3 Y705 phosphorylation in glioblastoma cells, perhaps by blocking access of a tyrosine phosphatase (35). Interestingly, mono-ubiquitination of another NH_2_-terminal lysine residue (K97) of STAT3 was implicated in recruitment of BRD4, a component of the activated positive transcriptional elongation factor (P-TEFb) complex, and induction of anti-apoptotic genes in HepG2 cells (33). Lastly, methylation of an arginine residue (R31) by PRMT2 was reported to attenuate leptin-induced STAT3 activation in the hypothalamus (27). However, the role of arginine methylation as a regulatory feature of STAT3 is controversial and perhaps cell type-specific (54).

#### Redox-Sensitivity and Oxidation

Signal transducer and activator of transcription 3 has 14 highly conserved cysteine residues. Nine of these residues are reported to be redox-sensitive and to control STAT3’s transcriptional activity by inhibiting Y705 phosphorylation or DNA binding, inducing formation of higher order complexes (dimers and tetramers), or by interfering with the function of the TAD (Table 2) (55–61). In general, oxidative stress inhibits canonical STAT3 transcriptional activity. Although mitochondria are a major source of ROS, it is not known if the redox sensitivity of STAT3 contributes to its mitochondrial activity, either enhancing or inhibiting those actions.

**Table 2:** **Redox-sensitive cysteines of STAT3**.

Residues (domain)	Function
C259 (NTD)	Responsible for cell lysis- and peroxide-induced dimers (57)
C328 (DBD), C542 (LD)	Glutathionylation blocks JAK2-mediated Y705 phosphorylation (56)
C418/C426/C488 (DBD), C765 (TAD)	Oxidation decreased DNA binding, SIE/c-*fos* promoter activity, and cell proliferation. Contribute (in various combinations) to peroxide-induced tetramer formation (59)
C418 (DBD), C712/C718 (TAD), C765 (TAD)	Responsible for Prx2-induced dimers and tetramers (58)

*DBD, DNA-binding domain; LD, linker domain; NTD, N terminus domain; TAD, transcription activation domain*.

Thiol targeting agents were reported to inhibit IL-6-induced STAT3 activation and increase its glutathionylation in HepG2 cells (55). These agents decreased nuclear accumulation of STAT3 and impaired expression of STAT3-target genes. S-glutathionylation of C328 and C542 within the DNA binding and linker domains, respectively, were shown to block JAK2-mediated phosphorylation of recombinant STAT3 Y705 likely due to steric or conformational reasons (56). Peroxide was found to induce STAT3 homodimer formation in HEK293 cells and a cysteine in the amino terminus was implicated (57). Cysteine residues within the DNA-binding domain and TAD appear to be especially crucial for STAT3 dimer and tetramer formation (58). Another study identified 3 redox-sensitive cysteines in the DNA-binding domain and one in the TAD (59). Oxidation of these cysteines by peroxide decreased STAT3 binding to an SIE *in vitro* and *in vivo* and IL-6-mediated reporter expression. Interestingly, evidence was reported that ROS may differentially affect STAT3 binding to DNA depending upon the nucleotide sequence of the promoter element, suggesting that the profile of genes activated by STAT3 may be modulated by oxidative stress (59).

We found that STAT3 activation in cardiac myocytes is impaired by glutathione (GSH) depletion (60). Glutathione monoethyl ester, which is cleaved intracellularly to GSH, prevented STAT3 inhibition, as did the reductant *N*-acetyl-cysteine. Notably, STAT1 activation was unaffected by GSH depletion. We also found that pre-treatment of human endothelial cells, neonatal rat cardiomyocytes, or adult mouse cardiac myocytes with thiolate-targeting electrophiles inhibited STAT3 activation and blocked induction of inflammatory genes (61). These compounds enhanced STAT3 glutathionylation with diamide treatment in adult cardiac myocytes and decreased the levels of STAT3 detected by Western analysis under non-reducing conditions. Thus, the redox-sensitive cysteines of STAT3 affect its cellular activation and availability in cardiac myocytes. Moreover, we found that STAT3 in the heart is affected by oxidative stress in a disease state, as monomeric STAT3 levels were decreased under non-reducing conditions in the Gαq model of heart failure in a redox-sensitive manner (61). Whether STAT3’s transcriptional activity is similarly affected in human heart failure is not known.

Evidence was recently reported that the redox-sensitivity of STAT3 has physiological relevance as well in the context of redox signaling by cytokines involving hydrogen peroxide generation. Peroxiredoxin-2 was shown to associate with STAT3 in HEK293T cells in response to treatment with IL-6 or oncostatin M (58). This association resulted in the oxidation of multiple cysteine residues of STAT3 within the DNA-binding domain, linker domain, and TAD, subsequent higher order complex formation, and attenuation of STAT3-mediated gene expression.

S-nitrosylation is a recently reported redox-related posttranslational modification that inhibits STAT3 activation. In microglial cells, endogenous NO from iNOS or treatment with s-nitrosoglutathione inhibited STAT3-induced gene expression and proliferation (36). STAT3 was observed to be s-nitrosylated on C259, which inhibited JAK2-mediated Y705 phosphorylation. Whether STAT3 is s-nitrosylated in the heart under conditions where iNOS is upregulated, such as the ischemic and failing myocardium, has not been reported.

## Canonical STAT3 Signaling

### STAT3 and Cardioprotection: Main Signaling Pathways

**Figure 3:**
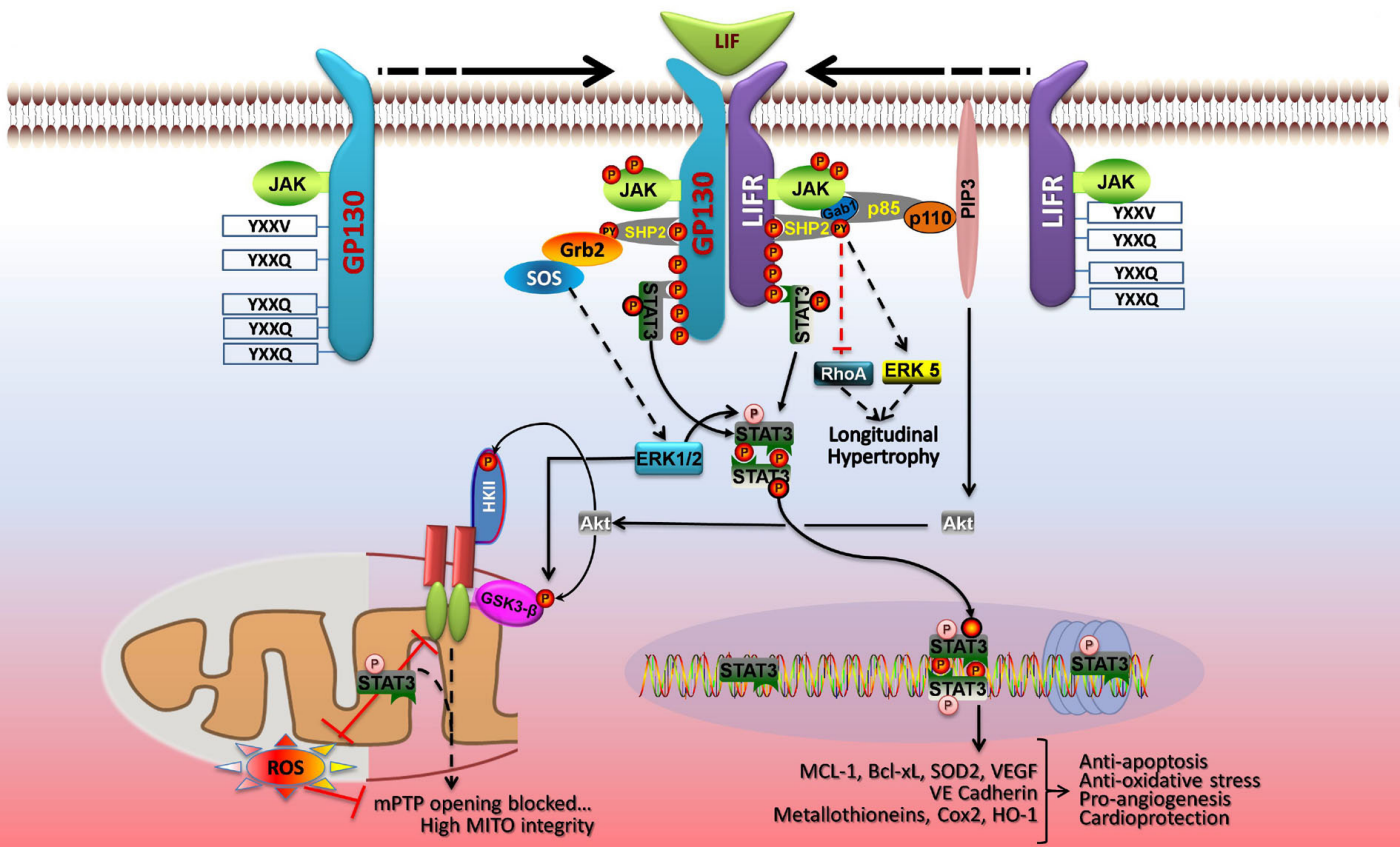
**LIF protects cardiac myocytes by both genomic and non-genomic means**. Dimerization of gp130 and LIFR activates the associated JAK tyrosine kinases, which phosphorylate recruitment sites for STAT3 and STAT1 (YXXQ), and a scaffold protein SHP2 (YXXV) that is coupled to ERK1/2, ERK5, and phosphoinositide 3-kinase (PI3K)-Akt activation. STAT3 and the SHP2-based signaling pathways are linked to cellular protection and/or cell growth. JAK-mediated STAT3 phosphorylation on Y705 enhances its dimerization and DNA binding. Activated STAT3 induces expression of anti-apoptotic, anti-oxidative stress, and pro-angiogenic genes, and STAT3 S727 phosphorylation boosts gene transcription. In the nucleus, U-STAT3 may affect gene expression either as a transcription factor or by modulating chromatin structure. In parallel, STAT3 has protective actions on mitochondrial function. STAT3 optimizes mitochondrial respiration and limits ROS formation, thereby opposing mPTP opening and ensuring mitochondrial integrity. These non-genomic actions of STAT3 are enhanced by S727 phosphorylation. ERK1/2 and Akt are components of the RISK pathway of cardiac protection that is evoked by pre- and postconditioning. ERK1/2 has protective effects on mitochondria *via* phosphorylation and inhibition of GSK3-α/β, which prevents mPTP opening. PI3K-Akt signaling also inhibits opening of mPTP *via* phosphorylation of GSK3-α/β or mitochondrial hexokinase II (HKII). See text for additional details. Adapted with permission from Zouein et al. (62).

Signal transducer and activator of transcription 3 signaling in cardiac myocytes in context is best illustrated by the induction of phosphorylation events downstream of LIF-induced dimerization of its membrane receptor LIFR with gp130 (Figure 3). Three major kinase cascades are activated with the central players being Akt, STAT3, and ERK1/2 that protect against oxidative or ischemic stress by acting on mitochondria or inducing gene transcription (62). LIFR and gp130 dimerization activates the associated JAKs, which phosphorylate recruitment sites for STAT3 (YXXQ) and a scaffold protein SHP2 (YXXV) linked to activation of ERK1/2, ERK5, and Akt (1, 62). These signaling pathways are linked to cellular protection and/or cell growth. STAT3 is activated by phosphorylation on Y705 by the JAKS, which leads to its dimerization and translocation to the nucleus. STAT3 is also phosphorylated by ERK1/2 on S727, which enhances its transcriptional activity (1, 3). Activated STAT3 induces expression of anti-apoptotic, anti-oxidative stress, and pro-angiogenic genes. STAT3 also optimizes mitochondrial respiration and limits ROS formation from complex I with ischemia, and these mitochondrial actions of STAT3 are enhanced by S727 phosphorylation (5). ERK1/2 couples to cell growth *via* an impact on transcription factors and intracellular increases in Ca^2+^, which activates calmodulin and the serine/threonine phosphatase calcineurin (not shown) (62). This in turn activates gene transcription (*via* NFAT) or de-represses cardiac gene expression *via* Ca2^+^/calmodulin-dependent protein kinases II (CamKII). ERK1/2 also has protective effects on mitochondria *via* phosphorylation and inhibition of GSK3-β, which prevents opening of mPTP (62). The phosphoinositide 3-kinase-Akt signaling network also inhibits opening of mPTP *via* phosphorylation of GSK3-β or mitochondrial hexokinase II (HKII) (62). Akt may also inhibit apoptosis and autophagy *via* phosphorylation of mTOR complex 1 (mTORC1), Bad, and Bax (not shown). Akt activation is linked to protective gene expression too.

### STAT3 in Acute and Chronic Cardiac Stress

#### Ischemia

Signal transducer and activator of transcription 3 has been implicated in protecting the heart from acute myocardial injury due to ischemia and ischemia–reperfusion (1, 3). STAT3 has also been implicated in the protection of the heart produced by various forms (ischemic, pharmacological, direct and remote) of pre- and postconditioning (3). For preconditioning, STAT3 has been implicated in both the early short-lived phase that does not involve gene expression and the delayed longer-lived phase that involves gene expression, known as the second window of protection (1). Many of these studies relied on JAK2 or STAT3 inhibitors, and thus the evidence in those cases implicating STAT3 is circumstantial.

Definitive evidence that STAT3 protects the heart from acute ischemic injury has been obtained from cardiac myocyte-targeted STAT3 knockout (KO) mice. Smith et al. reported that depletion of cardiac STAT3 did not affect infarct size; however, STAT3 was found to be crucial for preconditioning protocols (63). Hilfiker-Kleiner et al. also tested the role of STAT3 in ischemia–reperfusion and infarction using cardiac myocyte STAT3 KO mice (64). They observed greater infarct size and apoptosis 24 h after reperfusion in KO hearts with impaired fractional shortening after 7 days. Increased mRNA levels of the pro-apoptotic and pro-autophagy protein BNIP3 were observed 24 h after reperfusion, while mRNA levels of the prosurvival gene HSP70 were decreased. No changes were seen in VEGF expression, which is pro-angiogenic and previously linked to STAT3 overexpression or activation in the heart (65–67), as was the mitochondrial anti-oxidant protein SOD2 (68). STAT3 KO mice also exhibited a marked increase in mortality with myocardial infarction (MI). Using an inducible cardiac myocyte targeted STAT3 KO model, Bolli et al. reported that deletion of STAT3 eliminated the upregulation of antiapoptotic (e.g., Mcl-1, Bcl-xL, c-FLIP_L_, and c-FLIP_S_) and cardioprotective (COX-2 and HO-1) proteins normally induced in delayed preconditioning (69). Using a similar model, Enomoto et al. recently reported that STAT3 expression in cardiac myocytes contributes to remodeling during the subacute phase of MI (70). Deletion of STAT3 during days 11–24 after MI, resulted in worsened cardiac function and increased mortality. STAT3 ablation exacerbated cardiac fibrosis during the subacute phase of MI, along with an increased expression of fibrosis-related genes, presumably due to increased death of cardiac myocytes. Cardiac hypertrophy was enhanced by STAT3 ablation after MI and consequently capillary density was reduced in the border zone. Again, no change in VEGF expression was detected. Although the proper controls were performed, interpretation of these results may be complicated by the likely tamoxifen-induced cardiomyopathy that occurs in hearts expressing MerCreMer (71). Nonetheless, the study does support the conclusion that STAT3 has protective effects in the heart during the subacute phase post-MI. This conclusion is also supported by the results of a study that examined the impact of cardiac-specific deletion of SOCS3 on left ventricular remodeling after MI (72). SOCS3 is the intrinsic negative feedback inhibitor of STAT3 signaling. In SOCS3 KO mice, infarct size was markedly reduced 14 days after MI, as were myocardial apoptosis and fibrosis. Multiple protective signaling pathways were enhanced by SOCS3 deletion, including STAT3, as well as expression of antioxidants HO-1 and SOD2. Survival and cardiac function were improved. A similar finding of reduced myocardial injury after 24 h was observed with ischemia–reperfusion in SOCS3 KO mice and this was associated with increased expression of anti-apoptotic Bcl-2 family member myeloid cell leukemia-1 (Mcl-1), a gene target of STAT3 (73). In light of these findings, it is surprising that continuous activation of STAT3 in cardiac myocytes *via* a mutant gp130 receptor was associated with a worse outcome in subacute MI due in part to increased inflammation (74). The impact of concurrent signaling events in the later study on STAT3 signaling offers a possible explanation (75).

#### SAFE Signaling

Two major signaling pathways have been identified as contributing to the infarct-sparing effect of pre- or postconditioning. The Reperfusion Injury Salvage Kinases (RISK) pathway was identified first and entails activation with reperfusion of pro-survival kinases extracellular regulated kinase 1/2 (ERK1/2) and Akt that converge on mitochondria to decrease mPTP opening (76). The Survivor Activating Factor Enhancement (SAFE) pathway is a “RISK-free” mechanism of cardiac protection that was originally linked to the activation of TNF during reperfusion and the induction of JAK–STAT3 signaling by TNF receptor-1 (TNFR-1) (76). The relative contribution and importance of the RISK and SAFE pathways to myocardial protection can vary with the experimental ischemic protocol (pre- vs. postconditioning, direct vs. remote), as well as species. Some studies have identified a role for SAFE signaling in the initiation of the RISK pathway in protecting the heart by pre- and post-conditioning, although the basis for this is not defined (7, 77, 78). Given their clinical relevance, it is of note that SAFE activation is critical for protecting the heart by both remote pre- and postconditioning in larger mammalian species, such as pigs and humans, while RISK signaling is dispensable (76, 79). Interestingly, in humans STAT5 and not STAT3 is involved in remote ischemic preconditioning (80).

Besides, TNF, other agonists have been implicated in SAFE signaling, including sphingosine-1-phosphate, insulin, and IL-10 (7, 81, 82), and possibly SDF-1α (83). SAFE signaling protects the heart by both transcriptional and non-transcriptional means. Concerning the latter, the downstream targets are not well characterized, but may include direct STAT3-mediated suppression of NF-κB activity (84), phosphorylation of pro-apoptotic Bad protein (85), or inhibition of mPTP opening (86, 87). Ischemia or ischemia–reperfusion is reported to induce the translocation of STAT3 to mitochondria in heart by undefined means (88, 89), supporting the conclusion that the direct mitochondrial actions of STAT3 contribute to the protection afforded by SAFE signaling; although, this has not been definitely demonstrated.

#### Pregnancy

The heart is more vulnerable to ischemia–reperfusion injury in late pregnancy, and this is associated with impaired activation of protective signaling, including STAT3 (90). In addition, mice with cardiac myocyte-targeted STAT3 deletion develop a form of peripartum cardiomyopathy (PPCM) (91), a life-threatening condition that affects previously health women in the last month of pregnancy or the first months after giving birth. In hearts of STAT3 KO mice, pregnancy was found to be associated with increased oxidative stress due to blunted SOD2 expression (92, 93). This in turn leads to increased cathepsin D expression and activity, which generates a cleaved anti-angiogenic and pro-apoptotic 16 kDa form of the nursing hormone prolactin that impairs the vasculature thereby compromising cardiac myocyte metabolism. Serum levels of activated cathepsin D and 16 kDa prolactin are elevated in PPCM patients as well (92). The relevance of these findings to the etiology of human PPCM is uncertain, however. While titin gene mutations were found to be common in families with both peripartum and dilated cardiomyopathies, no STAT3 mutations were identified in the PPCM cases that were examined (93). On the other hand, left ventricular STAT3 protein levels are decreased in patients with end-stage heart failure due to PPCM (91).

#### Aging

Two lines of evidence support the importance of STAT3 in the heart with aging. First, cardiac STAT3 levels are reduced with age in various cell types and tissues, including the rodent heart (8, 9), but whether cardiac STAT3 levels are reduced with advanced age in humans is not reported. Second, mice with postnatal cardiac myocyte-targeted STAT3 KO encompassing exons for the DNA-binding domain develop heart failure with age (64). This is attributed in part to increase miR-199a-5p levels that were associated with increased asymmetric dimethylarginine (ADMA) synthesis, which in turn was linked to cardiac endothelial dysfunction, capillary loss, and potentially myocardial ischemia (94). Evidence suggests that STAT3 functions as a direct repressor of the promoter for miR-199a-5p and with total STAT3 KO in the adult mouse heart there was found to be a massive increase in levels of miR-199a-5p, which was linked to disturbance in the ubiquitin-proteasome system (UPS) due to suppression of ubiquitin conjugating enzymes (Ube) Ube2i and Ube2g1. Besides increased ADMA synthesis due to increased PRMT-1 levels, suppression of Ube2i and Ube2g1 in cardiac myocytes *in vivo* and *in vitro* was associated with downregulation of a- and β-myosin heavy chain (MHC) and derangement of sarcomeres. However, adult STAT3 KO hearts display normal levels of α- and β-MHC and SERCA2a (64), suggesting a potentially greater impact of increased ADMA levels to the pathology of the STAT3 KO hearts *in vivo* than sarcomere disruption.

#### Summary

Signal transducer and activator of transcription 3 is protective to the heart under acute ischemic conditions via canonical means involving transcription of anti-oxidative, anti-apoptotic and pro-angiogenic genes. In addition, STAT3 has poorly understood actions in protecting the heart from ischemia that may involve non-canonical actions in mitochondria (see below). Evidence also indicates that STAT3 is protective to the heart under chronic stress conditions, such as hypertension, pregnancy, and advanced age. The protective role of STAT3 in hypertension-induced cardiac hypertrophy has not been well studied, but in addition to involving transcriptional events likely involves non-canonical interactions with NF-κB (see below). In pregnancy and with aging, STAT3 in cardiac myocytes has been shown to be a critical determinant of endothelial function and health, although by different mechanisms: induction of protective anti-oxidant genes is important in pregnancy, whereas loss of STAT3-mediated repression of miR-199a expression may contribute to endothelial dysfunction in the heart with aging. The mechanism by which STAT3 represses gene expression in the heart is not known, as is the question of whether gene repression and induction by STAT3 involve distinct posttranslational regulatory mechanisms.

## Non-Canonical STAT3 Signaling

### U-STAT3 and Chromatin Remodeling

There is evidence that STAT3 in the heart regulates different sets of genes by two basic means: canonical (phosphorylated on Y705 and S727) and unphosphorylated STAT3 (U-STAT3) (1). In canonical signaling, Y705 phosphorylation favors formation of STAT3 *parallel dimers* that tightly bind to GAS elements in promoters and induce gene expression *via* assembly of an enhanceosome. Canonical signaling is associated with both inflammation (74) [although STAT3 has anti-inflammatory actions *via* non-canonical means (95)] and the protection from acute ischemic stress by upregulation of cardioprotective and anti-apoptotic proteins, including COX-2, HO-1, Mcl-1, Bcl-x_L_, c-FLIP_L_, and c-FLIP_S_ (69, 72, 73). Phosphorylation of STAT3 on S727 is thought to enhance canonical transcription by increasing recruitment of the histone acetyltransferase p300 (1, 44). S727 phosphorylation has also been implicated in the recruitment of tyrosine phosphatases, thereby attenuating canonical STAT3 signaling (49, 50).

U-STAT3 (*aka* latent STAT3) arises from canonical STAT3 signaling-induced STAT3 expression (96). U-STAT3 levels (with no Y705 or S727 phosphorylation) were shown to increase in nuclei of mouse hearts overexpressing the AT1 receptor and in neonatal rat ventricular myocytes treated with angiotensin II (97). U-STAT3 levels correlated with the degree of hypertrophy and U-STAT3 was postulated to induce a subset of inflammatory and pro-hypertrophic genes in the heart (97), although definitive evidence for this was not provided. It has been proposed that U-STAT3 serves as a means to prolong the inflammatory response initiated by canonical STAT3 signaling (96).

Some of the genes activated by U-STAT3 respond to its complex with unphosphorylated p65 subunit of NF-κB, and binding is not to GAS elements, but to a specific type of κB element in DNA that supports both binding of p65 homodimers and cooperatively with U-STAT3 (98). STAT3 S727 phosphorylation is not required, at least for the interaction of U-STAT3 with the p65 subunit (98). The role of S727 phosphorylation in U-STAT3-induced gene expression has not been systematically evaluated, although evidence reported to date indicates that S727 phosphorylation is not important (97, 98). In this regard, U-STAT3 is generally used to refer to non-tyrosine phosphorylated STAT3 without specifying the phosphorylation status of S727.

Nothing is known about how U-STAT3 activates the remaining target genes that do not require NF-κB (96). Presumably, these genes are activated by binding of U-STAT3 to a GAS or GAS-like element. Recently, acetylation of STAT3 on K685 was found to be required for the expression of most U-STAT3-dependent genes, with K685 acetylation having only a minor role in the expression of genes by tyrosine phosphorylated STAT3 (42). K685 may be important for the recruitment of p300 and/or K685 acetylation may facilitate stable STAT3 dimer formation in the absence of Y705 phosphorylation.

The possibility that U-STAT3, which tends to form *anti-parallel dimers*, also acts as a dominant negative mutant protein cannot be discounted (97). Moreover, the pathophysiological relevance of the AT1 overexpressing heart is uncertain, as adult cardiac myocytes express low levels of AT1 receptors and the hypertrophic actions of angiotensin II on the adult heart are attributable to increased blood pressure and not a direct effect on cardiac myocytes (99). Whether U-STAT3 accumulates in heart nuclei in other models of cardiac hypertrophy will need to be assessed.

Unphosphorylated STAT3 can bind GAS elements as a monomer or dimer, but binding is much weaker than seen with STAT3 phosphorylated on pY705 (100). Remarkably, U-STAT3 (non-tyrosine phosphorylated STAT3) binds more strongly to AT-rich DNA sequence sites and sequences common in S/MAR DNA elements that are implicated in chromatin organization (100). U-STAT3 also recognizes specific DNA structures, such as DNA nodes and four-way junctions that are involved in nucleosomal structure and assembly. These observations suggest that non-tyrosine phosphorylated STAT3 influences chromatin organization. Consistent with this possibility is the observation that deletion of the Drosophila STAT homolog Stat92E disrupts heterochromatin integrity and allows for transcriptional activation of genes that are not direct targets of Stat92E (101). Additionally, evidence indicates that Stat92E interacts with heterochromatin protein 1 (HP1) to regulate histone 1 (HI) and histone 3 (H3) function (102, 103). This epigenetic role of Stat92E is disrupted by its tyrosine phosphorylation and subsequent translocation of Stat92E to target genes (102). Evidence was recently reported that STAT5A functions similarly in several human cancer cells to maintain nucleosomal structure (104). Notably, STAT3, like Stat92E and STAT5A, possesses a conserved pentapeptide motif (PxVxI) for the binding of HP1. The question of whether STAT3 is important for heterochromatin stability in cardiac myocytes will need to be addressed, especially under stress conditions such as cardiac hypertrophy or heart failure.

Consistent with an extra-transcriptional role for STAT3 in the nucleus is the observation that the nuclei of various cell lines and primary cells contain substantial levels of U-STAT3 under non-stimulated conditions, i.e., ~40% of total cellular STAT3 (105, 106). Nuclear import of both U-STAT3 and tyrosine phosphorylated STAT3 is an energy requiring process involving the nuclear shuttle proteins, importin-α3 (-α6 in testes) and -β1 (106–109). Tyrosine phosphorylated STAT3 may also bind importin-α5 and -α7 (110, 111). A region (amino acids 150–162) within the coiled-coil region of STAT3 is critical for its recognition by importin-α3 and constitutive STAT3 nucleocytoplasmic trafficking (106). For tyrosine phosphorylated STAT3, but not U-STAT3, R214/215 in the α-helix 2 region of the coiled-coil domain is also required for nuclear import (111, 112). For either tyrosine phosphorylated or unphosphorylated STAT3, dimer formation (parallel and antiparallel, respectively) is not required for nuclear uptake (106, 113). Tyrosine phosphorylation does not affect STAT3 nuclear import; however, nuclear accumulation of STAT3 is determined by DNA binding, which is enhanced by tyrosine phosphorylation. Consequently, STAT3 S727 phosphorylation, which enhances recruitment of a tyrosine phosphatase, attenuates STAT3 nuclear accumulation (50).

Nuclear export of activated STAT3 is dependent upon its association with the class I histone deacetylase HDAC1, which in turn requires NH_2_-terminal acetylation of STAT3 (31). Brasier’s group has proposed that the binding of HDAC1 to STAT3 exposures one or more of the three nuclear export sequences (NESs) of STAT3 to the exportin CRM1. The nuclear export of stimulated and non-stimulated (latent) STAT3 is in part mediated by CRM1 (108, 113, 114). Two of the three NESs of STAT3 may be involved in constitutive export, while the third may be important for poststimulation export (115). In addition, there is some suggestion that U-STAT3 may shuttle between cytoplasm and nucleus independent of NLSs or NESs (113, 116). Neither nuclear import nor export of STAT3 has been well studied in cardiac myocytes.

### Interactions of STAT3 with NF-κB

Signal transducer and activator of transcription 3 and NF-kB signaling pathways interact at multiple levels in the cytosol, nucleus, and at various promoters. Activated (tyrosine phosphorylated and acetylated) STAT3 has been shown to interact with canonical NF-κB signaling, as well as with the non-canonical NF-kB pathway (1, 98, 108). In many cases, STAT3 serves to promote the survival and pro-growth actions of NF-kB activation. For instance, (1) in several human cancer cell lines phosphorylated Y705 and acetylated K685 STAT3 was implicated in the processing of NF-κB p100, which has pro-apoptotic and anti-oncogenic function, to the anti-apoptotic and oncogenic protein p52 (117); and (2) the enhanced nuclear NF-κB p65 levels that are frequently observed in cancer cells was attributed to p65 acetylation brought about by the binding of p65 to activated STAT3 in association with p300 (118). Evidence was reported that the opposite scenario occurs in the heart. IL-10, *via* activation of STAT3 signaling, was found to inhibit NF-κB-mediated hypertrophic and inflammatory gene expression by blocking NF-κB p65 activation in the cytosol (95). Exactly how this occurs, and whether inhibition is direct or indirect was not resolved. A conundrum for cardiac STAT3 signaling is why some cytokines that activate this protein, such as IL-6 are pro-inflammatory, while others such as IL-10, are anti-inflammatory (119). The temporal pattern of STAT3 activation and the consequent impact that would have on its interaction with NF-κB signaling may provide an explanation (75).

### STAT3 and Mitochondria

In 2009, the seminal observation was reported that STAT3 is present in the mitochondria of mouse hearts and loss of STAT3 is associated with mitochondrial dysfunction (46). Numerous reports subsequently established that STAT3 is found in mitochondria of various cell types and tissues and mitochondrial STAT3 has functional consequences. Approximately 30% of these reports involve cardiac myocytes or hearts and an equal number are cancer-related. In many cases, loss of STAT3 was accompanied by reduced activities of respiratory complexes I and II (4, 5). In mouse Stat3^−/−^ pro-B cells, deficient activities of complexes I and II were restored by expressing mutant STAT3 proteins with a phosphorylation refractory Y705F residue and either an intact S727 or a phosphomimetic S727 residue (46). A STAT3 protein unable to bind DNA or form dimers restored complex activities, while a STAT3 with a phosphorylation refractory S727A residue was ineffective. Thus, S727 phosphorylation is an important determinant of the mitochondrial actions of STAT3. Consistent with this conclusion are reports that S727 phosphorylation of mitochondrial STAT3 is required for the oncogenic growth of cancer cells (47, 48, 120–122). Which kinase or kinases are responsible for mitochondrial STAT3 S727 phosphorylation in cardiac myocytes is undefined, although the ERK pathway was found to be necessary for S727 phosphorylation of mitochondrial STAT3 and Ras-mediated transformation in human cancer cells (47). Whether other residues of STAT3 are phosphorylated and important for its mitochondrial actions is unknown, or whether STAT3 undergoes other posttranslational modifications in mitochondria such as acetylation.

In some cases, loss of STAT3 was associated with increased mitochondrial ROS production by undefined means (1, 4), but it is not reported whether loss of STAT3 enhances mitochondrial ROS in the heart. A few studies reported that loss of STAT3 impairs mitochondrial membrane potential, but this has not been systematically investigated (1, 4). Mitochondria from hearts of mice with postnatal cardiac myocyte-targeted STAT3 KO have enhanced calcium-sensitivity of mPTP opening (87). Although STAT3 was reported to co-IP with pore component cyclophilin D from rat heart mitochondria (87), it is unlikely based on stoichiometry that STAT3 opposes mPTP opening strictly by this mechanism (123). Decreased mitochondrial membrane potential due to reduced complex I activity may partially explain increased calcium-sensitivity of mPTP opening. Recently, MLS-STAT3E mice with cardiac myocyte-specific overexpression of mitochondria-targeted STAT3 with a mutation in the DNA-binding domain were generated (88). Inexplicably, MLS-STAT3E expressing mitochondria have a modest decrease in complex I activity; however, these mitochondria have a normal membrane potential and are protected against ischemic damage to complex I and release of cytochrome c into the cytosol. Compared to wild type mitochondria, ischemia does not enhance ROS production by MLS–STAT3E mitochondria (88).

Damage to complex I occurs during ischemia and leads to enhanced ROS formation during reperfusion that may further damage complex I (124, 125). Activity of complex I and/or other complexes is also reported to be reduced in various animal models of cardiac hypertrophy and heart failure, as well as human cardiomyopathies and heart failure (126–129). In these cases, mitochondrial dysfunction and enhanced ROS formation occur as well, but the importance of mitochondrial STAT3 to protecting mitochondrial respiration and integrity in chronic stress situations of the heart is not well studied.

The functional consequences of impaired complex I activity in cardiac hypertrophy and heart failure are not established; however, defects in complex I likely have significance beyond impaired ATP synthesis. As mentioned, complex I contributes to mitochondrial membrane potential and regulates mPTP opening (130–132). As NADH dehydrogenase, complex I is important in NAD^+^/NADH redox balance (131). Recently, deficiency of complex I activity due to KO of the Ndufs4 gene, which encodes a critical subunit of complex I, was reported to accelerate the progression to heart failure in the transverse aortic constriction (TAC) mouse model (133). Complex I deficiency correlated with an increase in NADH levels, which was associated with an increase in mitochondrial protein acetylation that was postulated to result from an inhibitory action of NADH and/or loss of NAD^+^ on SIRT3 activity. Pathological cardiac hypertrophy is associated with reduced intracellular NAD^+^ levels (134), and reduced SIRT3 activity could feed forward to further impair and cause damage to complex I. SIRT3 is positively linked not only to electron transport chain (ETC) and tricarboxylic acid (TCA) cycle activities (135, 136), but mitochondrial anti-oxidant defenses by increasing SOD2 activity (137) and maintaining mitochondrial GSH levels (138). We recently reported that hearts of SIRT3-deficient mice produce more ROS and show greater dysfunction when mitochondria are stressed as with a high fat diet (139). Others have shown that SIRT3 KO mice go into heart failure more rapidly with TAC (140) and are more susceptible to ischemia–reperfusion injury (141). In addition, in post-infarction heart failure in diabetic rats, reduced SIRT3 expression was associated with increased mPTP opening and acetylation of its activator, cyclophilin D (142). Thus, by maintaining complex I activity, STAT3 may preserve SIRT3 activity and mitochondrial integrity.

Complex I is also essential for respirasome (i.e., supercomplex) assembly in mitochondria (143). Decreased respirasomes leading to greater ROS has been proposed to drive the progression to heart failure (144, 145). Moreover, unincorporated complex I generates superoxide responsible for damage to mitochondrial DNA and the lipids and proteins of the inner membrane, including components of the ETC, that occurs in heart failure (145). Recently, increased levels of the matrix arm of complex I that are not incorporated into a fully assembled complex were linked to increased ROS production and aging in the mouse (146).

**Figure 4:**
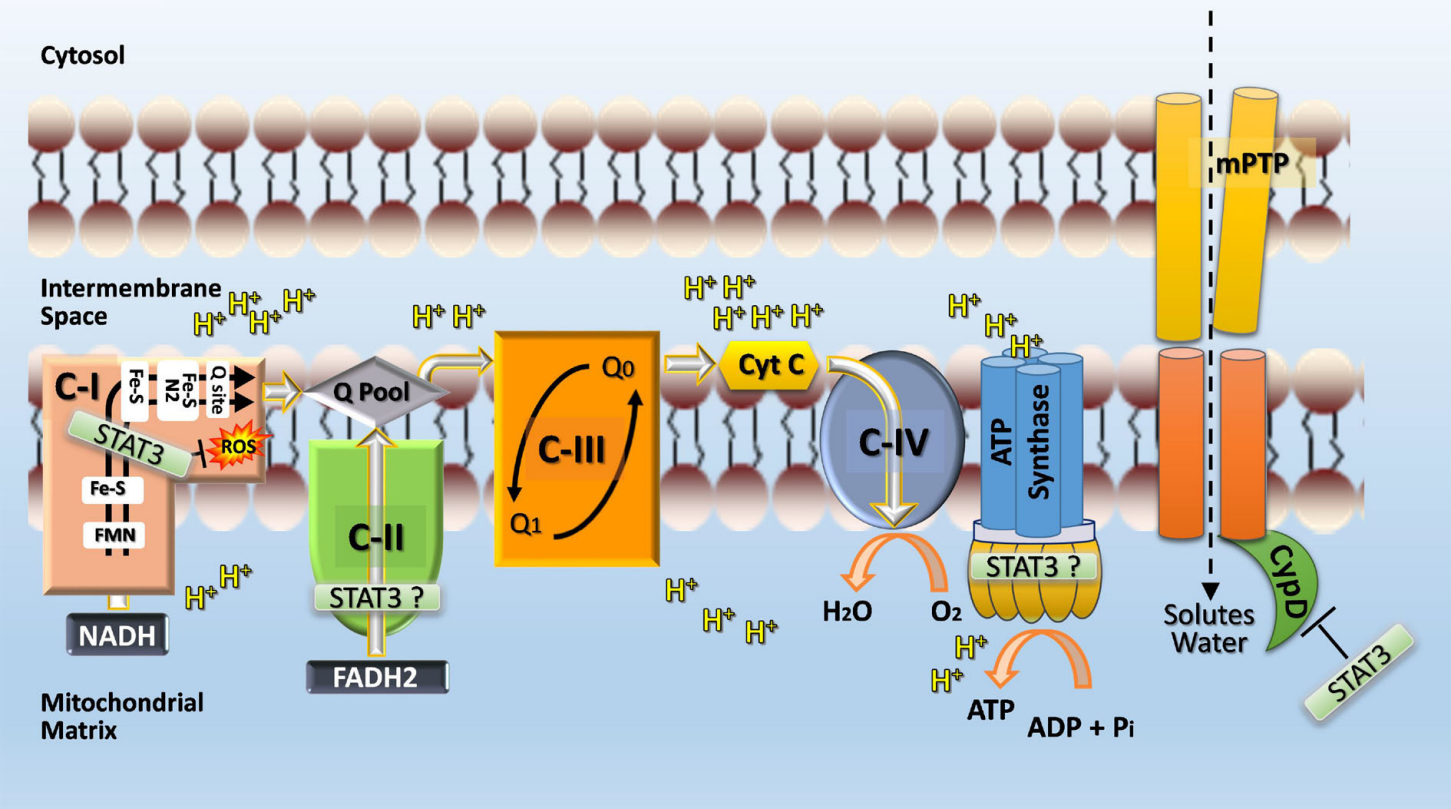
**Postulated role of STAT3 in mitochondria**. STAT3 may function directly or indirectly (e.g., by serving as a scaffolding protein for the posttranslational modification of a subunit protein) as a rheostat to ensure complex I function and prevent excessive ROS production, which would feedback and cause damage to complex I. STAT3 may also interact with cyclophilin D and thereby prevent opening of the mitochondrial permeability transition pore (mPTP) and subsequent disruption of mitochondrial integrity and function through the influx of solutes and water. Additional sites within the ETC where STAT3 may have positive functional actions, include complex II and ATP synthase (complex V). A possible engagement of STAT3 with complex V is postulated based on the observation that complex V activity is markedly reduced with STAT3 loss in Ras-transformed cells (120) and recent evidence identifying mPTP as the c-subunit of the F_1_F_O_ ATP synthase (147).

The mechanism by which STAT3 protects complex I and modulates mitochondrial metabolism is not known (Figure 4). The reported greater abundance of complex I/II to STAT3 in cardiac myocytes suggests that STAT3 affects mitochondrial function indirectly for example by serving as a scaffold protein that facilitates the posttranslational modification of some other protein (1). However, STAT3 was detected within complex I of liver mitochondria by Western immunoblotting suggesting that STAT3 may be further processed within mitochondria (46). STAT3 is an established binding partner of GRIM19 (complex I subunit B16.6 or NDUFA13), a critical supernumerary protein of complex I (45, 51). Association of GRIM19 with STAT3 occurs through the C-terminus of STAT3 and is positively affected by S727 phosphorylation. Uptake of STAT3 by isolated rat heart mitochondria is mediated by GRIM19, enhanced by STAT3 S727 phosphorylation, and requires mitochondrial membrane potential and energy (45).

GRIM19 is the only hydrophobic protein in the hydrophilic portion of the matrix arm of complex I (148, 149). Single-particle electron cryomicroscopy of bovine heart complex I places GRIM19 in the heel region of complex I, extending from the inner mitochondrial space into the matrix arm as far as NDUFS8 and spanning the inner mitochondrial membrane alongside ND1 (150). GRIM19 is reported to interact with NDUFA9 located in the transition region of complex I that anchors the matrix arm to the membrane and which undergoes a conformational change with hypoxia (151, 152). GRIM19 also is reported to interact with NDUFS3 and NDUFS4, which would place it close to the ubiquinone-reaction chamber formed by NDUFS2 and NDUFS7 (151, 153–156), a source of ROS during ischemia (157, 158). GRIM19 is important for both assembly of complex I and maintaining mitochondrial membrane potential (151, 159, 160). These functions occur independently of each other and are mediated by distinct regions. The region important for maintaining membrane potential (70–100) spans a predicted α-helix (74–98) and is adjacent to the conserved site of GRIM19 phosphorylation [T113] (151, 159, 161). Deletion of residues 70–80, 90–100, or the entire C-terminus of GRIM19 reduces mitochondria transmembrane potential and enhances sensitivity of mPTP opening (159). The importance of GRIM19 T113 phosphorylation is not known, but loss of S250 phosphorylation in another supernumerary subunit of complex I (NDUFA10) was recently shown to result in reduced ubiquinone reduction by complex I and mitochondrial depolarization (162). NDUFA10 is also located close to ND1 and ND3, which form a tunnel for ubiquinone access to the reaction chamber (154–156, 162). The role of STAT3 in GRIM19 phosphorylation is not known.

Based on the interaction of STAT3 with GRIM19, STAT3’s importance to complex I activity, and the impact of both GRIM19 and mitochondria-targeted STAT3 in preserving complex I integrity and membrane potential, it may be postulated that STAT3, *via* interaction with GRIM19, contributes to stabilizing the junction region between the matrix and membrane arms of complex I (48). This would ensure effective coupling of the matrix arm, involved in electron transfer from NADH to ubiquinone, to the membrane arm involved in proton pumping. Effective coupling may also limit electron leakage and ROS production from complex I, as the N2/ubiquinone binding site is a potential source of ROS (157). In fact, evidence derived from MLS-STAT3E mitochondria places the contribution of STAT3 to complex I activity downstream of the FMN moiety and in the vicinity of the N2/ubiquinone site, as there is a lack of ischemia-induced ROS in MLS–STAT3E mitochondria whereas NADH-ferricyanide reductase activity is unaffected (88). Consistent with this scenario are recent observations that breast cancer cells expressing mitochondria-targeted (MLS) STAT3 with a S727A mutation exhibit slower tumor growth, decreased complex I activity, and increased ROS accumulation under hypoxia compared with cells expressing MLS–STAT3 (48). On the other hand, cells expressing phosphoserine mimetic MLS-STAT3 S727D show enhanced growth and complex I activity, and decreased ROS production.

Mammalian complex I exists in two states, active (A) and de-active (D) (163–165). The state of complex I is determined by availability of substrates, divalent cations, and temperature. The A-form predominates over the D-form when sufficient substrates are available (NADH and ubiquinone) and at physiological temperatures. The A-form spontaneously converts into the D-form when substrate concentrations are reduced. Under normoxic conditions, 5–15% of complex I is in the D-form. Ischemia causes low substrate availability and favors the D-form, but the D-form reactivates into the A-form when substrates become available during reperfusion. The molecular mechanism behind the A/D transition is not known; however, involvement of three complex I subunits in the region of the ubiquinone binding site is proposed: 39 kDa accessory subunit NDUFA9 and mitochondrial encoded subunits, ND3 and ND1 that are involved in proton pumping (152, 166). The region around the matrix loop of ND3 is involved in conformational changes that occur during the A/D transition and selective fluorescence labeling and proteomic analysis on bovine heart mitochondria revealed that C39 of ND3 is exposed only in the D-form (152). Exposed C39 can be s-nitrosylated under hypoxic conditions, arresting complex I in the D-form (152, 164, 167). A recent study showed that s-nitrosylation of C39 of ND3 can be exploited to protect the heart from ischemia–reperfusion injury (168). Treatment of mice with a mitochondria-targeted nitrosylatng agent (MitoSNO) at reperfusion reduced ROS production, oxidative damage, and tissue necrosis. The explanation offered for this protection is that prolonging the time complex I spends in the D-form dampens the burst of respiration that occurs during reperfusion, thus limiting overall ROS formation from downstream sites in the ETC (164, 168). Notably, the D-form of complex I itself produces more ROS than the A-form and is more susceptible to ischemic damage (165, 169). Given the ability of STAT3 to reduce ROS formation from complex I, we assessed whether the A/D transition was deficient in complex I of MLS–STAT3E mitochondria; however, the A/D transition was clearly not affected by overexpressing mitochondrial STAT3 based upon functional measurements (Drs. Chen and Lesnefsky, unpublished observation).

The protective actions of STAT3 on respiration may involve other scenarios. Recently, mitochondrial-targeted STAT3 was shown to be required for the protective actions of prohibitin 1 against TNF-induced mitochondrial stress and apoptosis of cultured intestinal epithelial cells (170). Prohibitin 1, an abundant protein of the mitochondrial inner membrane, helps maintain mitochondrial structure and function and is required for optimal activity of complexes I and IV. STAT3 was found to interact with prohibitin 1 and this interaction was dependent on S727 phosphorylation. In activated CD4^+^ T cells, mitochondrial STAT3 (in this case, only pY705 STAT3 was assessed, but likely pS727 was involved as well) was implicated in the formation of ETC supercomplexes downstream of IL-6 (171). Formation of the supercomplexes resulted in mitochondrial hyperpolarization that was uncoupled from ATP production by oxidative phosphorylation and mitigated ROS production. Mitochondrial hyperpolarization allowed for increased mitochondrial calcium uptake and consequently higher cytosolic calcium that drove the expression of certain cytokines linked to inflammation during late activation of these T cells.

#### Summary

The non-canonical actions of STAT3 involve gene induction by U-STAT3 that may sustain a hypertrophic and inflammatory gene program in the heart in response to hypertension; however, definitive evidence for that conclusion is lacking. In addition, U-STAT3 or non-tyrosine phosphorylated STAT3 may have a role in genomic stability in the heart particularly under stress conditions. Although STAT3 is also known to interact with NF-κB in non-cardiac cells by various means, the importance if this interaction to the heart is not well studied. Evidence that STAT3 attenuates the contribution of NF-κB to hypertrophy and inflammation in the heart due to pressure overload would suggest that STAT3 activation could be exploited to protect the heart from hypertension-induced cardiac remodeling. Finally, STAT3 has been implicated in regulating mitochondrial respiration and limiting ROS production from the ETC. However, the molecular basis for the direct protective actions of STAT3 on mitochondria is not established. The finding that hearts of mice overexpressing a mitochondrial targeted STAT3 are protected from ischemia–reperfusion injury provides some hope that the mitochondrial actions of STAT3 could be exploited therapeutically.

## Conclusion and Future Directions

Much has been revealed about the role of STAT3 in the heart under acute stress such as ischemia and ischemia–reperfusion, and chronic stress such as pregnancy and aging. There is a general consensus that STAT3, in response to external stimuli, acts as a multifaceted protective protein by maintaining cellular homeostasis and antioxidant defenses. The activation and recruitment of STAT3 to induce both genomic and non-genomic activities is highly regulated by multiple posttranslational modifications, such as phosphorylation, acetylation, methylation, mono-ubiquitination, and oxidation. The co-ordination and integration of these posttranslational modifications in the heart in response to external stimuli is not well defined. Interestingly, non-stimulated STAT3, in the form of U-STAT3, has important genomic functions that are not well understood. In the heart, U-STAT3 may upregulate a specific sets of genes independently or *via* interaction with other transcription factors such as NF-κB, sustaining presumably an inflammatory and pro-hypertrophic effect. U-STAT3 may function as well in chromatin organization and assembly. While there is growing consensus for the importance of a non-genomic role of STAT3 in mitochondria, the molecular basis by which STAT3 modulates ETC activity and ROS production is not known, nor how that may affect mitochondrial redox status. All these actions of STAT3 raise a question as how to target it therapeutically to favor its beneficial actions in order to prevent or reverse adverse cardiac remodeling. Further investigation into how STAT3 is regulated is crucial to achieving this goal.

## Author Contributions

All authors contributed conceptually to the manuscript. All authors authored sections of the manuscript, contributed to the figure design, and approved the final version.

## Conflict of Interest Statement

The authors declare that the research was conducted in the absence of any commercial or financial relationships that could be construed as a potential conflict of interest.

## Abbreviations

ADMA, asymmetric dimethylarginine; AT1, angiotensin II type 1 receptor; Bcl-2, B-cell lymphoma 2; Bcl-xL, B-cell lymphoma-extra large; BMX, bone marrow kinase on chromosome X; BNIP3, BCL2/adenovirus E1B 19 kDa protein-interacting protein 3; BRD4, bromodomain-containing protein 4; CamKII, Ca^2+^/calmodulin-dependent protein kinases II; c-FLIP_L_, cellular FLICE-inhibitory protein, long; c-FLIP_S_, cellular FLICE-inhibitory protein, short; COX-2, cyclooxygenase-2; CRM1, chromosomal maintenance 1; DNMT1, DNA methyl transferase 1; EGFR, epidermal growth factor receptor; ERK1/2, extracellular-signal-regulated kinase 1/2; ETC, electron transport chain; EZH2, enhancer of zeste homolog 2; GAS, interferon γ (gamma)-activated sequence; GRIM19, gene associated with retinoic-interferon-induced mortality 19; GSH, glutathione; HDAC1, histone deacetylase 1; HO-1, heme oxygenase 1; HP1, heterochromatin protein 1; HSP70, 70 kilodalton heat shock proteins; IFNγ, interferon gamma; IL-10, interleukin 10; IL-6, interleukin 6; JAK1/JAK2, just another kinase 1 or 2; LIF, leukemia inhibitory factor; LIFR, leukemia inhibitory factor receptor; Mcl-1, myeloid cell leukemia-1; MHC, myosin heavy chain; MI, myocardial infarction; MLS, mitochondria localization sequence; mPTP, mitochondrial permeability transition pore; mTOR, mammalian target of rapamycin; ND1, NADH-ubiquinone oxidoreductase chain 1; ND3, (mitochondrially encoded) NADH dehydrogenase 3; NDUFA9, NADH dehydrogenase (ubiquinone) 1 alpha subcomplex subunit 9; NDUFS2, NADH dehydrogenase (ubiquinone) iron–sulfur protein 2, mitochondrial; NDUFS7, NADH dehydrogenase (ubiquinone) iron–sulfur protein 7, mitochondrial; NDUFS8, NADH dehydrogenase (ubiquinone) iron–sulfur protein 8, mitochondrial; NES, nuclear export signal; NF-κB, nuclear factor kappa-light-chain-enhancer of activated B cells; NFAT, nuclear factor of activated t-cells; NLS, nuclear localization signal; PAR-1, protease-activated receptor 1; PKC, protein kinase C; PPCM, peripartum cardiomyopathy; PRMT-1, protein arginine *N*-methyltransferase 1; PRMT2, protein arginine *N*-methyltransferase 2; P-TEFb, positive transcriptional elongation factor; RISK, reperfusion injury salvage kinases; ROS, reactive oxygen species; SAFE, survivor activating factor enhancement; SDF-1α, stromal cell-derived factor 1 alpha; SET9, histone-lysine *N*-methyltransferase SET9; SH2, Src homology 2; SHP2, Src-homology 2 domain-containing phosphatase 2; SIRT1, sirtuin 1; SIRT3, sirtuin 3; SOCS3, suppressor of cytokine signaling 3; SOD2, superoxide dismutase 2, mitochondrial; STAT3, signal transducer and activator of transcription 3; TAC, transverse aortic constriction; TAD, transcription activation domain; TCA, tricarboxylic acid; TNF, tumor necrosis factor; TNFR-1, TNF receptor-1; Ube2g1, ubiquitin conjugating enzyme 2g1; Ube2i, ubiquitin conjugating enzyme 2i; U-STAT, unphosphorylated STAT3.
